# Organic Bioelectronics: Materials and Biocompatibility

**DOI:** 10.3390/ijms19082382

**Published:** 2018-08-13

**Authors:** Krishna Feron, Rebecca Lim, Connor Sherwood, Angela Keynes, Alan Brichta, Paul C. Dastoor

**Affiliations:** 1 Centre for Organic Electronics, University of Newcastle, Callaghan, Newcastle, NSW 2308, Australia; Connor.Sherwood@uon.edu.au (C.S.); Paul.Dastoor@newcastle.edu.au (P.C.D.); 2 Centre for Brain and Mental Health Research, University of Newcastle, Callaghan, Newcastle, NSW 2308, Australia; Rebecca.Lim@newcastle.edu.au (R.L.); Angela.Hoslin@newcastle.edu.au (A.K.); Alan.Brichta@newcastle.edu.au (A.B.)

**Keywords:** bioelectronics, organic electronics, biocompatibility, neural interface, drug delivery, nerve cell regeneration

## Abstract

Organic electronic materials have been considered for a wide-range of technological applications. More recently these organic (semi)conductors (encompassing both conducting and semi-conducting organic electronic materials) have received increasing attention as materials for bioelectronic applications. Biological tissues typically comprise soft, elastic, carbon-based macromolecules and polymers, and communication in these biological systems is usually mediated via mixed electronic and ionic conduction. In contrast to hard inorganic semiconductors, whose primary charge carriers are electrons and holes, organic (semi)conductors uniquely match the mechanical and conduction properties of biotic tissue. Here, we review the biocompatibility of organic electronic materials and their implementation in bioelectronic applications.

## 1. Introduction

Bioelectronic devices can already be found in many applications in the medical sector. Indeed, medical electronic devices are now a mature technology. Examples include deep-brain stimulations to treat Parkinson disease [1], neural stimulation to treat epilepsy or paralysis [2], cochlear and vestibular implants for hearing and balance [3,4], and retinal prosthetic devices to treat blindness or vision loss [5]. As bioelectronics develops still further, broader applications such as controlling electrical appliances by neuronal read-out [6] become viable propositions.

While electronics such as sensors and actuators are a mature technology, the main challenge for bioelectronics remains in creating a stable communication pathway between the nervous system and electronic devices. The most common materials currently used to interface between biological tissue and conventional inorganic electronic materials are hydrogels driven by their low Young’s modulus of elasticity and electrical conductivity [7]. However, hydrogels are not semiconductors, which limits their use in bioelectronics. On the other hand, inorganic electronic materials have been conventionally used in bioelectronics due to a well-established integrated circuit industry and the wide range of inorganic semiconductor devices that are available. However, these abiotic electronic materials have significant drawbacks when it comes to forming a lasting interface with biotic living tissue due to their mechanical rigidity [8], surface structure [9], nature of charge transport [10], biofouling/surface oxides [11], and the limited number of materials that are biocompatible [12].

A promising new strategy, however, is to take advantage of the unique properties of organic semiconductors [13,14]. This review focuses on the biocompatibility of organic electronic materials and their potential use in bioelectronic devices. Organic conductors have the benefit of being mechanically flexible [8], have easily modifiable surface structure [9,15], and possess mixed ionic and electronic charge transport [10,16] and ease of processing [17], as summarised in Table 1. The mechanical and charge transport properties of organic semiconductors have been discussed at length [7,10]. In short, conducting polymers are soft solids with tunable surface roughness and a Young’s modulus ranging from 20 kPa to 3 GPa, which is much closer to the modulus of living tissue (~10 kPa) than inorganic (semi)conductors (~100 GPa). Importantly, the soft nature of organic semiconductors is thought to reduce inflammation due to the reduced strain between tissue and bioelectronic implant [18]. In addition, organic (semi)conductors can facilitate both electronic and ionic charge transport mechanisms, thus providing the ideal interface for transduction between the biotic and abiotic worlds [9].

Critical to the success of bioelectronics is reducing the immune response of an organism to the external device. Ideally, an implant is biologically inert and does not activate an immunological response, but allows target cells to integrate with the bioelectronic application. If the bioelectronic device elicits an immunological response, the device may become encapsulated within fibrous tissue, compromising or seriously disrupting the interface between device and neural tissue. Therefore, before building an implantable device, the biocompatibility of each component needs to be tested. Here, we review the types of biocompatibility tests that are frequently used, the outcome of these tests for various organic semiconductors, and identify classes of organic semiconductors that are of interest to bioelectronic applications.

## 2. Biocompatibility

In addition to electrical and mechanical properties, biocompatibility is essential for bioelectronic devices. However, biocompatibility is not uniquely defined and a biomaterial can elicit different responses depending on the local tissue environment. As such, ubiquitous materials that are completely biocompatible in all biological environments remain a theoretical concept. Consequently, the term ‘biocompatibility’ should only be used in the context of the material’s application environment. Indeed, Spector et al. define ‘biocompatibility’ as ‘a condition met by a biomaterial or medical device usually based on the tissue response elicited by an implant in an animal model’ [19]. Nevertheless, when assessing novel biomaterials, designing new bioelectronic devices, or conducting fundamental research, the end-application and its specific target tissue may not be known. Consequently, it is important to state the type of tissue and which assay was chosen for biocompatibility tests clearly. The International Organization of Standardization (ISO) has established a series of tests and evaluations to examine the biocompatibility of a material (ISO 10993; Biological evaluation of medical devices). The type of tests required for a material is dependent on the type of body contact (surface contact, external communicating devices or implant devices) and the length of contact time. Once this has been established, the biocompatibility of the material is assessed by tests of cytotoxicity, sensitization, irritation, systemic toxicity (acute), sub-acute and sub-chronic toxicity, genotoxicity, hemocompatibility, chronic toxicity, carcinogenicity, reproductive/developmental toxicity, biodegradation—identification and toxicokinetic studies, physicochemical, morphological, and topographical characterization, and immunotoxicology [20].

Testing for these responses can take place in in vitro, ex vivo, or in vivo environments, however ethical and financial constraints often dictate the use of in vitro cell culture systems during initial stages of testing new materials or devices. In vitro experiments allow for the assessment of the material’s toxicity to cells, cell adhesion, cell activation, cell death, structural stability and preservation of electronic functionality of the biomaterial in cell culture media at 37 °C. Testing for functionality depends on the biomaterial’s intended purpose. For example, testing could involve simple conductivity measurements before and after culture media exposure or testing the device while immersed in cell culture media without exposure to living tissue [14].

Implantation of a bioelectronic device inserted in a living host can interact with tissue in four ways [21]:

- *Toxic*: biomaterial has adverse effects on surrounding tissue e.g., cell death, immunological response, organ failure and inflammation.
- *Bioinert*: non-toxic, biologically inactive. The material has no or minimal interaction with the living host. However, an adverse response may still occur as fibrous tissue may encapsulate the device, thus loosening and then severing the interface of the device with target cells resulting in device failure.
- *Bioactive*: material is non-toxic and biologically active. The device forms an intimate connection with the host tissue.
- *Bioresorbable*: non-toxic material dissolves in the host tissue. The bio-electronic device only functions temporarily. The surrounding host tissue can eventually replace the synthetic device.

In vitro assays in the field of organic bioelectronics are often conducted over an ‘acute’ period of up to 10 days [22]. In contrast, in vivo testing usually occurs over a longer ‘chronic’ period which may be weeks, months, or years [3,5,23]. Long-term issues and potential remedies associated with implantable devices are best studied in vivo. For example, silicon causes a chronic in vivo response to encapsulate the foreign object, which progressively increases the impedance of the electrode–tissue interface, ultimately leading to complete isolation of the electronic device from the target tissue [24,25,26,27] even though in vitro tests using silicon appear favourable [28,29]. In comparison, Kung et al. showed that the introduction of an organic semiconductor, poly(3,4-ethylenedioxythiophene (PEDOT), resulted in a nerve interface that was stable for over 7 months in vivo [23]. To date, it has been unclear if other organic (semi)conductors show similar favourable long-term stability. It should be noted that in vivo experiments are generally only undertaken after encouraging in vitro results have been obtained [30]. As such, in vitro assays only provide an initial screening test for organic electronic materials with long-term clinical testing required after development of the biomaterial or bioelectronic device has sufficiently progressed. For the evaluation of biocompatibility of materials and medical devices for clinical and commercial purposes, well-established international standards as described in ISO10993 and ASTM F series must be consulted [31]. Furthermore, agencies such as the United States Food and Drug Administration who approve the use of specific devices should also be consulted before end-user applications are developed [10].

In vitro cytotoxicity tests can be categorised into three types of assay: direct contact, agar diffusion, and extract dilution [31]. While agar diffusion potentially compromises device functionality and extract dilution (elution studies) are limited by maintaining cell culture conditions, the most appropriate test for organic semiconductors is direct cell contact. Contact of either primary cells and (immortalised) cell lines with organic semiconductors have been used for direct contact cytotoxic assays. Cytotoxicity tests determine if a biomaterial contains harmful extractables that result in death or damage of isolated cells. A series of tests are typically used to examine cytotoxicity and include; mitochondrial dehydrogenase performance measurement (MTT assay), XTT (tetrazolium dye) cell proliferation assay, neutral red uptake cytotoxic assay, and colony formation cytotoxic assay. These cytotoxic tests assess cell damage by morphological means, and take measurements of cell damage, cell growth, and indicators of cell metabolism (ISO 10933-5).

## 3. Cell Adhesion

**Figure 1:**
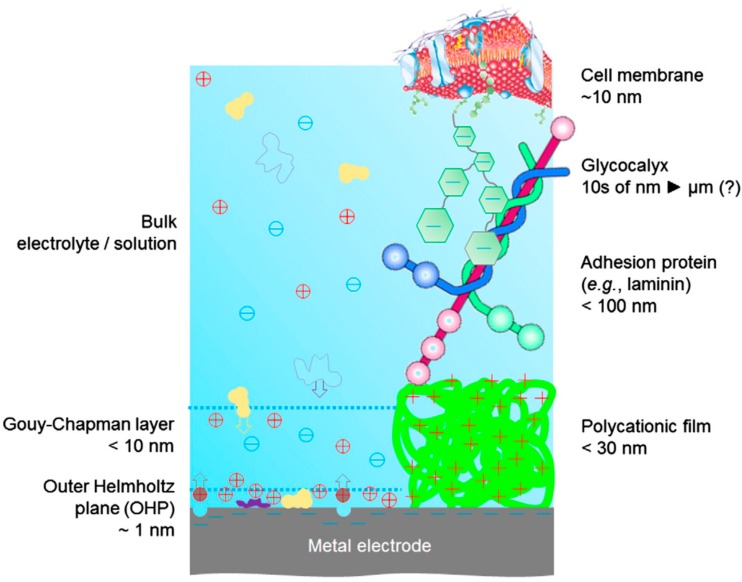
Reprinted with permission from [34] under the terms of the CC BY license.

For useful neural bioelectronics applications, the semiconductor material must allow adhesion and support for neuronal cells to establish intimate contact with living tissue. While many organic semiconductors are found to be biocompatible, cells will not directly adhere to all biocompatible polymers. Some organic materials, such as graphene and carbon nanotubes, were reported to have good cell adhesion [32,33]. However, many organic semiconductors are hydrophobic leading to poor spreading of the culture medium and cell adhesion. The glycocalyx (pericellular matrix) that surrounds the cell membranes is composed of a negatively charged network of proteoglycans, glycolipids, and glycoproteins [34]. Therefore, a common method of promoting adhesion is to exploit the electrostatic interaction between the extracellular surface charge and positively charged ionic polyamino acids, such as poly-l-lysine, poly-d-lysine and poly-l-ornithine, as depicted in Figure 1. While these adhesive layers have been shown to be very effective in in vitro conditions, they have significant drawbacks. Some adhesive layers suffer from long-term instability. For example, poly-l-lysine is digested by certain cell types, while poly-d-lysine is less easily digested but still prone to adhesion failure [34]. The adhesive layer may also modulate or interfere with the interaction between the organic (semi)conductor and cells thus compromising the functionality of the intended bioelectronic device. [10,35]. To address this potential issue, Bonetti et al. synthesised lysinated quaterthiophene [36]. The lysine end groups enabled good adhesion of cells directly to the (semi)conductor without the need for an additional adhesion layer. The semiconductor was shown to be both biocompatible and functional. The ability to modify organic semiconductors in this manner allows for the fabrication of more simplified and stable organic bioelectronic devices. Wettability and cell adhesion can also be improved using simple plasma treatments [37]. Cell adhesion to PEDOT-based biomaterials is generally good [38,39,40] although further adhesion improvements can be achieved by binding functional groups (peptides) to the polymer backbone [10,35]. Alternatively, modifying the way polymers are applied can also improve adhesion. For example, poly(ethylene glycol) and perfluorinated polyether have poor wettability for cell adhesion (too hydrophilic or hydrophobic respectively), and flat films of these materials show no cell attachment. However, if films are ‘micropatterned’ with these polymers, good cell adhesion was observed without the need for an additional adhesion layer [41]. Indeed, good cell adhesion was reported for 2,4-bis [4-(*N*,Ndiisobutylamino)-2,6dihydroxyphenyl] squaraine (a squaraine-based organic semiconductor), because it spontaneously forms a highly textured surface even when mixed with another semiconductor, e.g., phenyl-C61-butyric-acid-methyl ester (PCBM) [42]. Adhesion can also be a dynamic process since changes in the local chemistry due to cellular secretion or accumulation of metabolites can reduce or improve cell adhesion [34]. Smart surfaces with switchable adhesion [43] could provide an effective solution to biofouling or facilitate removal of temporary implants. Zhu et al. showed that conducting polymers can be made to specifically recognise and attach to target cells while at the same time being repelled by cells binding to other tissue [44]. This cell membrane-mimicking conducting polymer demonstrates the potentially high cell specificity of these polymers.

## 4. Organic Semiconductors for Bioelectronic Application

Polymers are frequently used in prosthetic devices, including naturally occurring polymers such as collagen, sodium alginate, and cellulose, used in devices such as heart valves (silicone), as well as synthetic polymers used for intraocular lenses (polymethylmethacrylate) and hip arthroplasty systems (polyethylene). Here, we focus on organic electronic materials that are relevant to bioelectronic devices. For example, Ghezzi et al. successfully recovered light sensitivity in blind rats using poly(3-hexylthiophene-2,5-diyl) (P3HT) as an artificial retina [13]. Khodagholy et al. recorded brain activity in rats and human epilepsy patients using poly(3,4-ethylenedioxythiophene) doped with poly(styrenesulfonate) (PEDOT:PSS) as a biointerface layer [38,45]. Kung et al. implanted PEDOT coated electrodes in the thigh of rats to record activity in the regenerative peripheral nerve [23]. Simon et al. used ion pump devices with PEDOT:PSS as the active component to deliver specific neurotransmitters to particular neuronal cells in guinea pigs [46]. Organic semiconductors that have been investigated for bioelectronic applications are summarised in Table 2.

### 4.1. Materials for Electroactive Scaffolds

**Figure 2:**
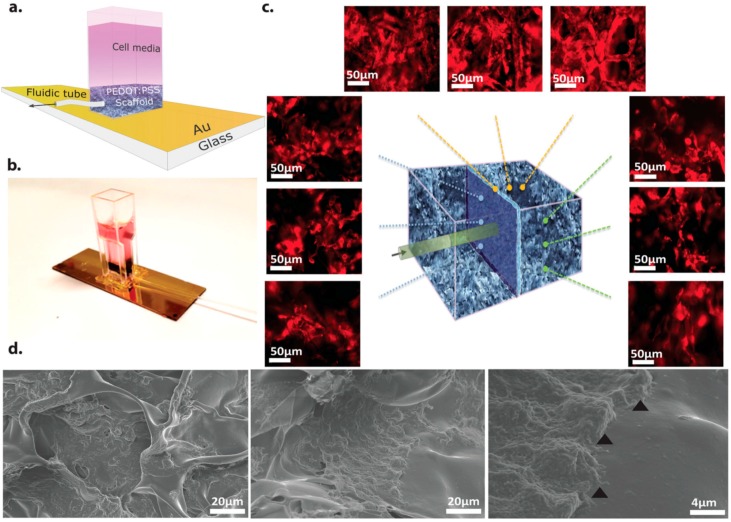
PEDOT:PSS bioelectronic 3D scaffold. (**a**) schematic; (**b**) a photograph of the 3D conducting polymer scaffold showing the gold (Au) coated glass slide (used to provide electrical contact with the scaffold) and the integration of the media perfusion tube within the plastic cuvette used to contain the media; (**c**) immunofluorescence images and illustrative diagram (centre); (**d**) scanning electron microscope (SEM) images. Reproduced with permission from [61]. Copyright 2017 by John Wiley and Sons.

Most in vitro experiments involve growing cells on planar films. The corresponding 2D growth of the cells is easily evaluated using optical techniques but does not represent the growth of cells in 3D in vivo environments. Therefore, research efforts are now focused on building 3D in vitro cell culture systems that closely resemble real tissues [58]. One approach involves fabricating scaffolds that promote cell growth in a 3D environment and there are a number of excellent reviews on passive materials for 3D support structures [59,60]. Here we focus on organic materials used for electroactive scaffolds, i.e., materials that are not only used as a support structures, but also provide an electronic function. Organic semiconductors that have good cell adhesion properties are ideal candidates for 3D scaffold structures, because they do not require an additional adhesion layer. Cell growth in 2D planar cell culture assays is typically monitored using optical techniques. However, interpreting optical data in a 3D scaffold is more challenging. An alternative is to use advanced tomography techniques, but these can only be accessed intermittently at specialised facilities and this is consequently not a preferred option for a cell culture lab requiring reasonable throughput. Electroactive scaffolds, however, could provide an indirect way of monitoring cell proliferation. For example, PEDOT:PSS was used to fabricate a macroporous scaffold to support 3D cell culture (Figure 2 [61]). The electronic properties of the electroactive scaffold were seen to change after seeding with cell culture, suggesting that the scaffold itself could be used to assay cell growth in situ. In addition, a porous PEDOT:PSS structure was used to electrically stimulate fibroblast cells enabling control over cell adhesion and cell secretion rates [62]. Thus, porous PEDOT:PSS scaffolds have the potential to both control and monitor cell growth and function.

**Figure 3:**
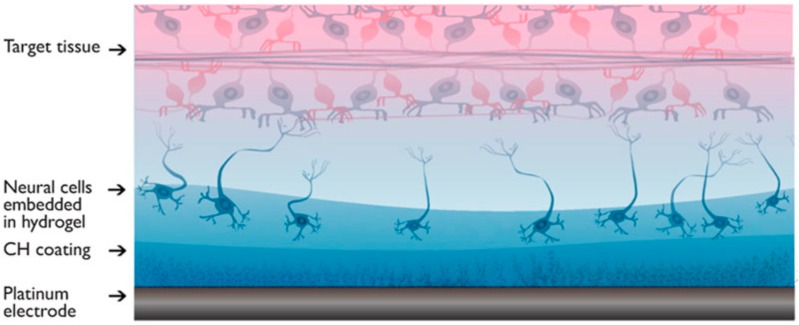
Living electrode consists of a typical platinum microelectrode covered by two layers of hydrogels. The bottom layer (blue) consists of a non-degradable conductive hydrogel loaded with PEDOT and optimised for electrical properties. The top layer (pink) is a biodegradable hydrogel loaded with and optimised for neural cell growth. Once good cell adhesion/growth is achieved this layer dissolves. Reproduced with permission from [68]. Copyright 2017 by Materials Research Society.

Instead of using a material that simultaneously fulfils both purposes, i.e., acting as a scaffold and providing electrical conduction, two or more separate materials can be combined to form a hybrid network. Hydrogels are commonly used as insulating scaffolds and have been combined with PEDOT and PPy [63]. Such electroconductive hydrogels have a large contact area, resulting in a low electrical impedance. For example, hydrogel scaffolds with electrochemically deposited PPy had an impedance of 7 kΩ at 1 kHz compared to 100 kΩ for a PPy film alone [18]. Recently, a conductive and biocompatible hydrogel combined with a polyurethane matrix, PEDOT:PSS, and liquid crystal graphene oxide was fabricated to facilitate electrical stimulation during 3D culture of human neural stem cells. Not only was high viability achieved with this type of hybrid scaffold, but electrical stimulation during culture was shown to enhance neuritogenesis [64]. An intimate mix of a conducting polymer with a hydrogel can be achieved as follows. A hydrogel is deposited on to a conducting polymer film. The hydrogel sample is then immersed in an aqueous solution of a monomer, followed by electropolymerisation to obtain a conducting polymer network that permeates throughout the hydrogel [65,66]. Such hybrid scaffolds can be further functionalised by the addition of biomolecules to influence cell adhesion and differentiation. For example, a PPy-chitosan hydrogel was synthesised to improve biological conduction and was used to better maintain heart function after myocardial infarction [67]. To further improve the electrical contact between neuronal tissue and electrodes, Goding et al. developed a ‘living electrode’ consisting of two layers of hydrogel scaffolds [68] as shown in Figure 3. One layer is immersed with PEDOT for good electrical conduction while the other is loaded with neuronal cells and optimised for cell growth. The layer optimised for cell growth is biodegradable and only functions to support cell proliferation and differentiation to produce neural networks in the short term. In the longer term, the neural network is expected to support itself based on its own extracellular matrix. This double layer approach allows separate optimisation of cell attachment and growth without compromising electrical conductivity. The impedance of the double layer hydrogel system is similar to a single PEDOT-loaded hydrogel coating and is superior to a bare platinum electrode.

Other organic conductors are fully biodegradable/bioresorbable and, therefore, have the potential to be used as temporary electroactive scaffolds and include aniline pentamer-based block co-polymers [56] and a pyrrole-thiophene based polymer [57]. To facilitate the biodegradability of conducting polymers, they are typically blended or synthesised as a composite with non-conductive biodegradable polymers such as polylactide, polycaprolactone, poly(lactide-co-glycolide), polycaprolactone fumarate, poly(lactide-co-polycaprolactone), polyurethane, chitosan, gelatin, collagen or heparin [69].

### 4.2. Materials for Neural Interface Electrodes

A neural interfacing system can have two potential functions (1) stimulation of a neuronal preparation and/or (2) readout of signals from transducing biotic and abiotic components. The readout from neural tissue usually manifests as a series of action potentials (APs). An AP is a rapid change in voltage due to activation of voltage-gated ion channels localized to the neuronal membrane. An AP is triggered when the membrane potential crosses a voltage threshold, which varies between neuron types, but is typically around −50 mV. A number of techniques are currently used to monitor AP activity in neuronal populations including whole-cell patch-clamp recordings, intracellular sharp recordings, and extracellular field recordings. Each of these techniques has inherent advantages and disadvantages.

Whole-cell patch-clamp recordings give excellent low-noise resolution of individual neuron activity with the ability to monitor microvolt changes in membrane potential. Recordings in this configuration allow quantitative and qualitative evaluation of AP characteristics including; rise time, amplitude, width, decay kinetics, and after hyperpolarization. However, since only one individual neuron can be recorded at any one time, data collection is time consuming. Similarly, intracellular sharp microelectrode recordings acquire data from individual neurons but with more noise and less resolution than whole-cell patch clamp techniques. However, sharp microelectrode techniques cause less disruption of cellular contents in the intracellular configuration than patch clamp electrodes that usually dialyse the cell contents. Therefore, sharp electrodes allow for longer recording time of AP discharge rates. The extracellular recording technique maintains the integrity of cellular contents since no cell membranes are breached but with lower resolution of AP characteristics. The advantage of the extracellular recording technique is that it allows the acquisition of AP data from more than one neuron at a time. For example, in-vivo extracellular recordings from hippocampal CA1 pyramidal cells detected APs from neurons within a 140 μm radius of the electrode, which suggests the activity of up to 1000 neurons could be simultaneously monitored [70].

More recently, population data has been recorded from multi-electrode array systems. These systems allow recording and stimulation of neural signals across multiple sites, simultaneously. Some systems are designed primarily for in vitro recording of cultured neurons, organotypic explant preparations, and acute brain and/or spinal cord sections, while others have the capacity for in vivo recordings in live animals—freely moving or anaesthetized. To maximise the spatial recording resolution of multi-electrode arrays, it is important to minimise current spread in the extracellular fluid and crosstalk between neighbouring electrodes, which is challenging given that electrical contact between electrode arrays and tissue is poor (high impedance) [3,5]. Since neurons of the same type generate identical action potentials, the only way to differentiate the activity of a single neuron is to reduce the distance and impedance between the electrode and the neuron of interest [71]. The contact between the sensing/actuating electrodes and tissue can be improved through the use of a conducting polymer. Conducting polymer coatings are thought to increase the effective contact area of the electrode with cells due to a more complex nanostructure, which ultimately results in a lower impedance and higher charge capacity [35].

PEDOT:PSS or other PEDOT derivatives are one of the most common organic electronic materials used for sensing or actuating electrodes [2,8,10,72]. PEDOT can be deposited using facile coating techniques such as spin coating, inkjet printing, roll-to-roll coating and electrodeposition. In order to pattern the PEDOT layer on a micrometre scale photolithography is the preferred method [45,73]. However, PEDOT:PSS dissolves and delaminates in an aqueous environment limiting its long-term stability unless cross-linkers are added to stabilise the polymer [74] or is electrochemically deposited [39]. The addition of cross-linkers, however, reduces the ion mobility of the biomaterial giving rise to a trade-off between stability and ion transport properties [11,75].

The electrode size and configuration required to record from individual cells, such as retinal ganglion neurons, is a technical challenge that has only recently been addressed with some success [5,38,45,74,76]. For example, ‘NeuroGrid’ consists of a network of electrodes with individual electrode size of 10 × 10 μm^2^ and interelectrode spacing of 30 μm. The interface of the electrode is composed of poly(3,4-ethylenedioxythiophene) doped with poly(styrenesulfonate) (PEDOT:PSS), which was shown to substantially decrease the electrical impedance mismatch between tissue and electrode [45]. The reduction in impedance at the bio-organic interface compared to a bio-inorganic interface was postulated to be due to the mixed electronic and ionic conductivity of PEDOT:PSS [74]. PEDOT:PSS indeed exhibits a high ionic mobility, which is advantageous in bio-interfacing [77]. However, conclusive evidence for this hypothesis is lacking, which means that the cause of reduced impedance is still unclear.

Poly(pyrrole) (PPy) is another water-soluble conducting polymer that has been used for neural electrodes in in vivo models [78]. A PPy coating was shown to decrease the impedance at 1 kHz from ~1 MΩ for bare gold to ~100 kΩ. Conductive polymers can be tailored to specific applications by doping with various molecules such as polystyrene-sulfonate (PSS) or sodium dodecylbenzenesulfonate (NaDBS) [79]. These films have also had nerve growth factors incorporated into them, which decreased the electrode impedance from ~100 kΩ to 15 kΩ [80]. PPy has been successfully functionalised with synthetic peptides to enhance nerve cell attachment [78]. For example, neurotrophin-3 (NT3) is a common neurotrophic growth factor that has been incorporated in PPy electrodes, via *para*-toluene sulfonate, to enhance the connection with cochlear nerve cells in guinea pigs [81]. Electrical stimulation was not impeded by NT3 even though the impedance of the PPy electrode was comparable to a bare platinum electrode. Multiple neurotrophins can be incorporated in conducting polymers and delivered simultaneously. Indeed the most vigorous neurite outgrowth from cochlear implants was seen when combining NT3 and brain-derived neurotrophic growth factor (BDNF) in PPy with pulsed electrical stimulation [82].

Currently, most organic neural interfaces make use of a metal grid, because of its superior conductivity. The organic (semi)conductor acts as an interface between the biotic and abiotic parts. However, Guo et al. recently showed that it is possible to make a fully organic multielectrode array using patterned PPy on poly(dimethylsiloxane) [72]. Both electrodes and leads were made of PPy and the device was devoid of metals. The device was fully flexible and successfully measured the neural activity in in vivo rat models.

Devices that are capable of both stimulation and recording offer the prospect of a dynamically self-regulating medical device for the treatment of epilepsy or similar neural disorders. A more sophisticated device is required to enable both stimulation and detection of APs. Benfenati et al. fabricated an organic field-effect transistor instead of a standard electrode and demonstrated that the same device was capable of bidirectional stimulation and recording of primary neurons [50]. P13 polymer was used as the organic semiconductor and gold as source and drain contacts. P13 also acted as a capping layer to avoid the exposure of gold contacts to the saline solution. Moreover, the device was transparent, thus allowing for simultaneous optical imaging of bioelectrical activity.

The long-term stability of neural interface electrodes with conducting polymer coatings requires more investigation. Currently there are few reports of long-term studies in the literature. One study has shown that a PEDOT-coated electrode implant consistently performed better than a stainless steel electrode over a 7-month period in a rat model [23].

A neural interface provides a way of transducing ionic signals into electronic signals. The capability of PEDOT and other conducting polymers to facilitate ionic and electronic transport also finds applications in neuromorphic circuits and processing, i.e., artificial computing systems that mimic neurobiological architectures. Indeed, a PEDOT:PSS based organic electrochemical transistor that mimics some of the spatiotemporal processing capabilities of a neurobiological system has been successfully implemented [83].

### 4.3. Materials for Photostimulation

**Figure 4:**
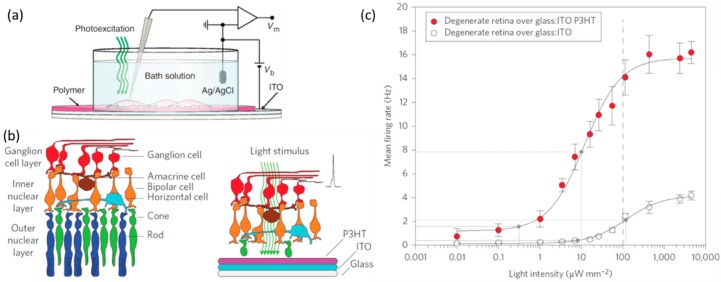
Diagram depicting (**a**) experimental setup and device structure of a polymer artificial retina, (**b**) the part of the retina that is replaced by the artificial device and (**c**) mean neural firing rate as a function of light intensity with and without an organic semiconductor. Reproduced with permission from [13,14]. Copyright 2011 by Springer Nature.

To fabricate bioelectronic devices capable of photo-stimulating neurons (artificial retinas) the biomaterial must not only be electronically, but also optically active. Moreover, to mimic the natural photoreceptors in the human eye, organic semiconductors with appropriate light absorption spectra are required. Semiconductors such as methyl-substituted ladder-type para-polyphenylene (MeLPPP) absorb in a similar region of the visible spectrum as human blue photoreceptors, poly(3-hexylthiophene-2,5-diyl) (P3HT) mimics green photoreceptors and violanthrone 16,17-bis(octyloxy)anthra[9,1,2-cde-]benzo[rst]pentaphene-5,10-dione mimics red photoreceptors [84]. While P3HT (green absorber) has been successfully applied to photostimulate a rat retina [85], MeLPPP (blue absorber) and violanthrone-79 (red absorber) have not yet been used in any bioelectronic devices. Combining three complementary organic semiconductors would enable artificial retinas capable of colour vision. Mixtures of P3HT:PCBM have been successfully used to fabricate an artificial retina and have also been demonstrated to photostimulate embryonic primary hippocampal neurons (Figure 4) [13]. The mixture of these two semiconductors (P3HT and PCBM) form a heterojunction, which is required to generate charge carriers efficiently [86]. Interestingly, the photostimulation device is believed to operate via a capacitive mechanism as opposed to a charge injection mechanism (resistive coupling). In other words, the photogenerated charge in the organic semiconductor layer modulates the membrane potential to at least the AP threshold, thus triggering neuronal firing. In this mechanism no charge is injected in the neuron or electrolyte solution. Hence, no electrolysis takes place and no hydroxide ions were detected [14]. Ghezzi et al. also fabricated P3HT retinas (i.e., without PCBM) and found that they were equally effective in neuronal photostimulation as P3HT:PCBM retinas [13]. Since the presence of PCBM does not affect the performance of the artificial retina, it would appear that efficient charge generation in the bulk of the photoactive layer is not required for neuronal photostimulation. Another study indicates that the measured transmembrane current is almost entirely explained by a capacitive current; suggesting that no ionic transport occurs through the ion channels in the cell membrane [42]. An exact quantification of the ionic and capacitive current contributions to the total transmembrane current is difficult to establish with standard voltage clamp techniques [87]. A photothermal mechanism was also ruled out since such a process could only account for a change in cell membrane potential of 1 mV, which is too small to be the sole trigger of action potentials [88]. Further investigation is necessary to better understand the photostimulation mechanism and this information is crucial for designing optimal bioelectronic devices for neuronal photostimulation.

A P3HT-based artificial retina was demonstrated to restore light sensitivity in blind rats [13,85]. Similarly, a P3HT:N2200 bulk heterojunction layer coated on a multielectrode array formed a photostimulation device that could elicit APs in immature chick retinas when exposed to light [22]. The potential advantages of these organic artificial retinas include ease of fabrication, patterning and no need for external power sources or connection cables. While optical requirements for an artificial retina are easily met due to the availability of a wide range of organic semiconductors, the challenge of achieving colour vision with a retinal prosthesis lies in combining and appropriate patterning of three organic semiconductors (red, green, and blue receptors) at a scale equivalent to the density of natural photoreceptors near the fovea, which is approximately 6000/mm^2^ [5,89]. The technical challenge of patterning such a device would be alleviated if only one semiconductor was required to provide colour vision. Gautam et al. showed that the polarity and temporal profile of a polymer photoactive layer (P3OT:N2200) depends on the colour of light [90]. Hence, it may be possible to achieve colour vision with a single photoactive layer through smart analysis of the temporal current profile. In addition, the first long-term study of a fully organic retinal prosthesis was recently reported [85]. The implant consists of three layers: a fibroin silk substrate, PEDOT:PSS, and P3HT. The visual acuity and behaviour of rats with and without the subretinal implant was monitored for 6–10 months. Both the increase in the basal metabolic activity in the primary visual cortex and vision-dependent behavioural studies showed that the implant effectively restored vision in blind rats.

### 4.4. Materials for Nerve Growth and Guidance

Conducting polymers such as PPy have the potential to improve nerve growth and guidance, which is crucial for nerve regeneration applications (e.g., bridging nerve gaps such as spinal cord injuries) and is essential for neural integration with implants. Micropatterning a PPy-based electrode can significantly influence the direction or polarisation of axon growth [91] without affecting axonal length. By contrast, Lee et al., using electrospun PPy-coated nanofibers, observed that electrical stimulation increased neurite length by 40–50% and neurite formation by 40–90% [92]. Moreover, aligned nanofibers resulted in longer and more neurites compared to randomly oriented fibers. PPy doped with polystyrene sulphate was also shown to be a suitable material to increase neurite length when electrically stimulated [48].

Indeed, Schwann cell migration and neurite extension from dorsal root ganglions were more proliferative on a PPy film compared to a glass substrate (control) [93]. During an in vivo nerve regeneration study, silicone tubes were used to bridge a 5 mm transection of rat sciatic nerve. The area of regenerated nerve, number of regenerated nerves, and their recovery rate was reported to be enhanced in PPy-coated silicon tubes compared to plain silicone tubes [93]. Further studies are required to determine the potential of PPy as a nerve guidance conduit.

In addition to the general promotion of cell growth, the targeted regeneration of specific tissue aids recovery from injury where certain tissue were destroyed. For example, to bridge a nerve gap, it is important that nerve cells (rather than only fibroblast cells) proliferate. Such targeted regeneration can be achieved using a 3,4-ethylenedioxythiophene (EDOT) based conducting polymer, which displayed high resistance to nonspecific cell binding while tissue specific neurite outgrowth was effectively enhanced (124% enhancement) [44]. These cell membrane-mimicking conducting polymers hold promise for targeted nerve regeneration applications.

A combination of both electrical and chemical (neurotrophic growth factors) stimulation is emerging as a preferred approach to improve neuron extension/growth [94]. For example, the neurite length of PC12 cells were longer when polypyrrole doped with chondroitin sulphate (PPy-CS) was functionalised with type I collagen compared to PPy-CS only, and longer still when simultaneously electrically stimulated [95].

Other than direct electrical stimulation, organic semiconductors can also be used to control the release of neurotrophic growth factors. For example, PPy doped with p-toluene sulphonate (PPy-pTS) was embedded with NT3. The rate of NT3 release from the PPy-pTS film could be increased by electrical stimulation [96]. Consequently, an electrode was fabricated with low impedance that encourages neurite outgrowth towards electrodes [97].

### 4.5. Materials for Drug Delivery

**Figure 5:**
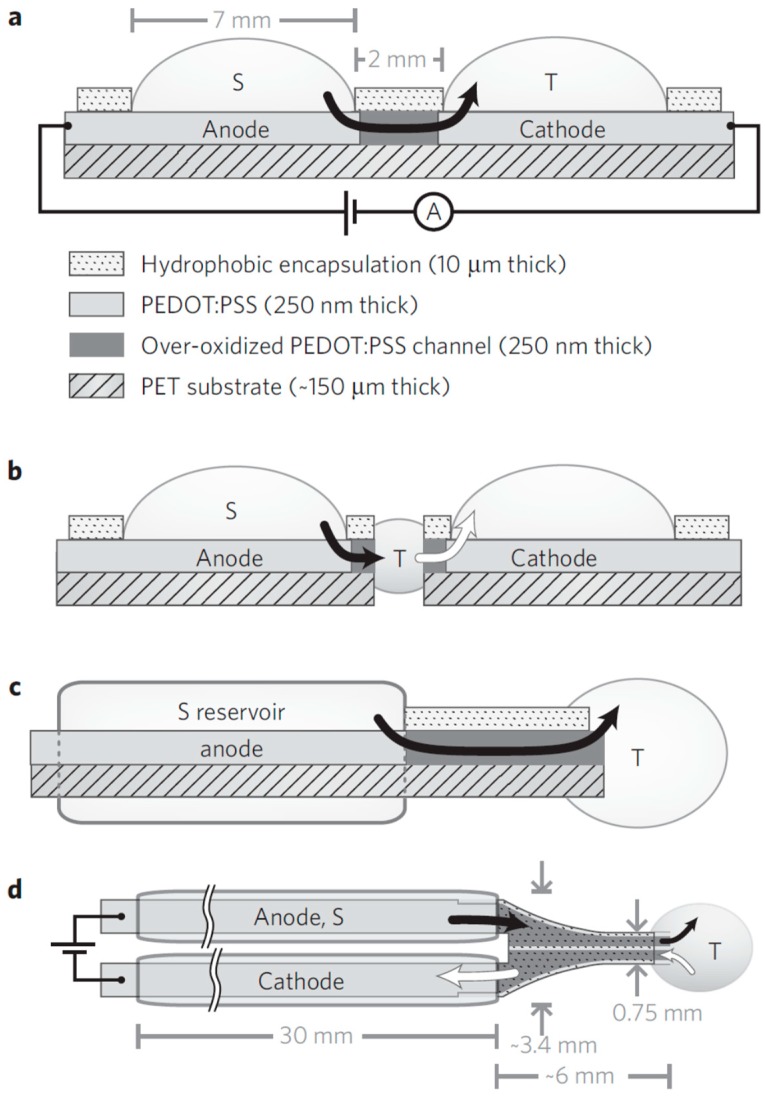
Diagram of a planar ion pump device. The black arrow indicates ion flow from the reservoir to the target area. (**a**–**c**) show side views and (**d**) shows a top view of the encapsulated device. Reproduced with permission from [46]. Copyright 2009 by Springer Nature.

The controlled delivery of biochemical agonists or antagonists would be of great advantage in therapies that require local drug delivery of small quantities at specific times [46]. Second and third generation drug delivery research aims to design delivery systems that are triggered by environmental parameters such as changes in pH, glucose content, or temperature, and systems that facilitate long-term, modulated, targeted delivery [98]. In this section, we focus on the use of polymers to release chemicals via electrical stimulation as opposed to continuous, passive leaching of a drug through a polymer. If delivery is sufficiently controlled, a neural interface could be achieved to deliver neurotransmitters and/or inhibitors to (de)activate ligand-gated ion channels. Berggren and co-workers developed PEDOT:PSS-based ion pump devices to deliver neurotransmitter in-vivo [46]. The device comprises two PEDOT:PSS electrodes connected via an over-oxidised PEDOT:PSS channel, as shown in Figure 5. One electrode is in contact with an electrolyte reservoir and the other with the target tissue. The over-oxidised channel is ionically conductive, but electronically insulating. When a voltage is applied across the electrodes, ion motion occurs from source to target regions via the PEDOT:PSS ion channel to compensate for the electronic current that flows in the external circuit. In this manner, controlled neurotransmitter delivery in the inner ear of a guinea pig was achieved and was demonstrated to stimulate cochlear cells [46]. Uguz et al. used a PEDOT:PSS-based ion pump to deliver a neurotransmitter on the surface of the cortex in a rat model to successfully alter neural behaviour [99]. The ion pump only required 0.5 V to elicit appropriate electrical activity; low-voltage operation is preferred to avoid unintended redox reactions. A neurotransmitter delivery rate in the order of 0.1–1 nmol/s was achieved when the device was switched on and was 10^−5^ nmol/s when it was switched off.

Another approach to drug delivery takes advantage of the structural changes that conducting polymers, such as PPy, undergo during redox reactions. The doped polymer can be electrically switched between the oxidised and reduced state, which is accompanied by the movement of hydrated ions in and out of the film [100]. The electrically controlled swelling and de-swelling of the film was previously used to fabricate electromechanical actuators [101,102]. Alternately, if biomolecules are used as dopants, they can be delivered via an electrochemical method, as demonstrated by Wadhwa et al. [100] who achieved controlled delivery of an anti-inflammatory steroid. Nerve growth factor [103] and antibiotics [104] have also been released using this method.

### 4.6. Materials for Biosensing

The compatibility of organic electronic materials with enzymes and other sensing elements has led to an increase in biosensing-related research activity in recent years [100]. While biosensors often do not necessitate implantation or direct interactions with the nervous system, they can be complementary to the aforementioned bioelectronic applications. For example, one can envisage an insulin delivery implant that is activated by a glucose sensor to manage diabetes. Blood glucose detectors are common while saliva-based glucose detectors are being developed at a rapid pace [105,106]. As biosensors are further developed, in vivo monitoring of cellular metabolism and early stage detection of disease become possible [107]. The key considerations for biosensors is the ability to use them for aqueous analytes (e.g., blood, saliva) without triggering any unwanted electrochemical reactions, analyte specificity and sensitivity. Furthermore, low power consumption [108], low-cost and facile fabrication are desired to maximize commercial potential. As such, the printability and low-cost nature of organic electronic devices is highly advantageous [109]. Indeed, biosensors have been successfully printed on flexible substrates [110,111]. Biosensors can be realized using organic semiconductors in a variety of thin-film transistor architectures. In general, a recognition element is used to detect a target biomolecule, which modifies the current–voltage characteristics of the thin film transistor. A calibration curve can be determined, thus enabling a quantitative biosensor. The recognition element may be an enzyme, antibody, nucleic acid, living cell or biopolymer [112]. The reader is referred to Elkington et al. [105] for a full review of suitable recognition materials for specific analytes. Biosensors rely on the (semi)conducting properties of organic thin films as well as the diffusion of analytes through grain boundaries [111]. As such, film morphology is important in many architectures. In addition to polymer inks, small molecules such as pentacene and sexithiophene are commonly used in biosensors for their high conductivity [111]. These small molecules are generally vacuum-deposited, which means they do not have the advantage of printability.

## 5. Conclusions

The field of biomaterials and tissue engineering are already accustomed to using polymeric materials due to their favourable mechanical properties and biocompatibility. On the other hand, electronics are largely based on inorganic materials that are mechanically incompatible with biotic tissue and often illicit cellular and immunological responses that can be either cytotoxic or bioinert in nature. Organic semiconductors have emerged as a class of material that combine the favourable qualities of both the biotic and abiotic world and are currently being applied to various bioelectronic applications including electroactive scaffolds, neurostimulation, nerve cell regeneration/guidance, and drug delivery. While bioelectronic devices based on (semi)conducting polymers have been successfully fabricated, and in many cases show better performance than their inorganic equivalent, a theoretical framework underlying the functional mechanisms is lacking. For example, the mechanisms underlying stimulation of semiconductors with light is not well understood. Electrical stimulation is also largely based on empirical results. Without advanced mechanistic models, device design rules can only be built up through empirical data, which slows down progress. Future challenges also involve long-term clinical studies to demonstrate that organic bioelectronics can provide tangible medical benefits. Organic bioelectronics devices are rapidly developing and hold great promise for next-generation medical technology. The potential benefits for the health sector warrants further study and investment.

## Figures and Tables

**Table 1:** Overview of material properties for abiotic, organic semiconductors and biotic living tissue. Adapted from [10]. Copyright Materials Research Society 2015.

Aspect	Abiotic Electronic Biomedical Devices	Conjugated Polymers	Biotic Living Tissue
Composition	Inorganic metals, semiconductors	Organic molecules, including functionalized polythiophenes, copolymers, and dopants	Complicated, dynamic mixture of water, electrolytes, proteins, lipids, nucleic acids
Physical State	Hard solids	Soft solids	Extremely soft solids
Morphology	Single crystal, polycrystalline, or amorphous	Semicrystalline or amorphous	Complicated and dynamic; cells, intercellular spaces
Surface structure	Nearly flat	Can be tailored from nearly flat to rough and fuzzy	Complicated and dynamic
Mechanics: Young’s modulus	~100 GPa	10 MPa–3 GPa (as solids) 20 kPa–2 MPa (as gels)	~10 kPa (cortex)
Charge carriers	Electrons, holes	Electrons, holes, and ions	Ions
Mass transport	Relatively limited at the molecular scale (solids), but can potentially incorporate microfluidic channels at large length scales	Facilitate ion transport with appropriate counterions, bicontinuous structures, deposition into hydrogels	Locally liquid-like biological environment

**Table 2:** Organic (semi)conductors with confirmed biocompatibility. For each material, the assay environment, tissue type investigated, and whether an adhesion layer was used, is shown. Note that not all (poly)(3,4-ethylenedioxythiophene) (EDOT/PEDOT), polypyrole (PPy) and poly(aniline) (PANI) variances are included as separate materials. The majority of PEDOT, PPy and PANI derived materials are biocompatible [47].

Material	Assay Environment	Cell Type	Cell Adhesion	Reference
poly(3-hexylthiophene-2,5-diyl), **P3HT**	Ex vivo, in vitro	Hippocampal neuron from embryonic 18-day rat embryos. Retinal neurons from 13–15 day chick embryos.	poly-l-lysine	[13,14,22,37]
phenyl-C61-butyric-acid-methyl ester, **PCBM**	Ex vivo	Hippocampal neuron from embryonic 18-day rat embryos	poly-l-lysine	[14,22]
Quaterthiophene, **T4**	In vitro	Primary dorsal root ganglion (DRG) neurons, postnatal Sprague Dawley rats	poly-l-lysine	[36]
Lysinated quaterthiophene, **T4Lys**	In vitro	DRG neurons, postnatal Sprague Dawley rats	Inherently good	[36]
2,4-bis [4-(*N*,Ndiisobutylamino)-2,6dihydroxyphenyl] squaraine, **DIBSq**	In vitro	N2A cells	Inherently good	[42]
Polypyrole, **PPy**	In vitro, ex vivo, in vivo	PC-12 cells, primary chicken sciatic nerve explants, subcutaneous and intramuscular sites, adult male Lewis rats	Poly-l-lysine	[48]
poly(3,4-ethylenedioxythiophene), **PEDOT**	In vitro	Primary cortical cells, embryonic (18–20 days) mice.	Inherently good	[49]
poly(3,4-ethylenedioxythiophene) doped with poly(styrenesulfonate), **PEDOT:PSS**	In vivo	Hippocampal and cortex neurons, male Long Evans rats	Inherently good	[45]
*N*,*N*′-ditridecylperylene-3,4,9,10-tetracarboxylic diimide, **P13**	In vitro	Dorsal root ganglion neurons, post-natal rat	Poly-d-lysine + laminin	[50]
**C_60_**	In vitro	Dorsal root ganglion neurons, mice	Poly-d-lysine	This work—Supplementary Information
poly(2,3-bis-(3-octyloxyphenyl)-quinoxaline-5,8-dyl-alt-thiophene-2,5-diyl), **TQ1**	In vitro	Dorsal root ganglion neurons, mice	Poly-d-lysine	This work—Supplementary Information
16,17-Bis(n-octyloxy) anthra [9,1,2-cde] benzo[rst]pentaphene-5,10-dione, **Violanthrone-79**	In vitro	Dorsal root ganglion neurons, mice	Poly-d-lysine	This work—Supplementary Information
**Nafion**	In vitro, In vivo	HEp-2 cells. Male ICR mice	Inherently good	[51]
Pentacene	In vitro	Neurons from forebrain of mouse embryos	Poly-l-lysine, laminin	[52]
Graphene	In vitro	Brain tissue from postnatal mice	Poly-l-lysine	[32]
Carbon nanotubes	In vitro	Hippocampal cells from Sprague Dawley rats	Inherently good	[33]
poly{[*N*,*N*′-bis(2-octyldodecyl)-naphthalene-1,4,5,8-bis(dicarboximide)-2,6-diyl]-alt-5,5′-(2,2′-bithiophene), **N2200**	In vitro	Retina from chick eyes at embryonic day 13–15	l-ornithin, laminin	[22]
Poly(aniline), **PANI**	In vivo	Subcutaneous implantation into male Sprague-Dawley rats beneath the dorsal skin	Inherently good	[53]
Ethylene-vinyl acetate, **EVAc**	In vivo	Subcutaneous implantation into male Sprague-Dawley rats beneath the dorsal skin	Inherently good	[53]
Polyethylene, **PE**	In vivo	Subcutaneous implantation into male Sprague-Dawley rats beneath the dorsal skin	Inherently good	[53]
poly(p-phenylenevinylene) derivatives, **PPV**	In vitro	AsPC-1, HMEC-1, BV-2 and C8-D1A cells	Inherently good	[54,55]
PLA-b-AP-b-PLA copolymer, **PAP**	In vitro	H9c2 cells	Inherently good	[56]
Pyrrole-thiophene based polymer, **BECP**	In vitro, in vivo	Human neuroblastoma cells, subcutaneous implantation into rats	Inherently good	[57]

## Supplementary Materials

Supplementary Materials can be found at http://www.mdpi.com/1422-0067/19/8/2382/s1.
