# Supplementary material for: Organic Bioelectronics: Materials and Biocompatibility
Source: Int J Mol Sci. 2018 Aug 13;19(8):2382. doi: 10.3390/ijms19082382 (PMC6121695; doi:10.3390/ijms19082382)
Supplement: Supplementary file 1 [file ijms-19-02382-s001.pdf]

Supplementary Information - Organic bioelectronics: materials and biocompatibility

Krishna Feron[*1], Rebecca Lim[2], Connor Sherwood[1,2], Angela Keynes[2], Alan Brichta[2], Paul C. Dastoor[1]

1 Centre for Organic Electronics, University of Newcastle, Callaghan, NSW, 2308, Australia

2 Centre for Brain and Mental Health Research, University of Newcastle, Callaghan, NSW, 2308, Australia

Preparation of semiconductor films

Three organic semiconductor films were prepared on glass coverslips. Violanthrone 79 (10mg/mL) and TQ1 (10mg/mL) were dissolved in chlorobenzene and limonene respectively to form inks. The inks were then deposited onto glass coverslips (22 x 22mm, thickness; 0.13 - 0.17 mm) via spin-coating at 1,000 rpm for 2 minutes (acceleration of 1596 rpm/s). The C_{60} semiconductor was deposited onto glass coverslips (dimensions as above) using an automated vacuum deposition process at a rate of 0.3A/s. After coating coverslips, the films were dried for a minimum of 2 hours. Coverslips coated with semiconductors and control glass (no film) were coated with poly-D-lysine (0.1mg/mL, Sigma-Aldrich) overnight. After coating with poly-D-lysine, coverslips were washed and bathed in with Dulbecco's phosphate buffered saline until plating.

Preparation of dorsal root ganglion cell cultures

All animal experiments were approved by University of Newcastle Animal Ethics Committee. Mice (C57Bl6) aged 7 days were used for these experiments. Mice were anaesthetised with ketamine (100 mg/kg) and then decapitated. The dorsal root ganglia (DRG) were dissected from the vertebral column in a cold HEPES buffered solution containing (in mM); 146 NaCl, 4.7 KCl, 0.6 $MgSO_4$, 1.6 $NaHCO_3$, 0.13 NaH_2PO_4, 2.5 $CaCl_2$, 7.8 glucose and 20 HEPES.

After dissection from the vertebral column, DRG neurons were dissociated in trypsin and collagenase (0.175 mg/mL and 0.125 mg/mL respectively). Dissociated DRG neurons were plated (4.7 x 10^4 cells/mL, 200µL per film) onto the organic semiconductor films in a medium containing; neurobasal A media (Thermo-Fisher; 86%); horse serum (Thermo-Fisher; 10%); PenStrep (Thermo-Fisher, 1%); L-glutamine supplement (Thermo-Fisher, 1%); B-27 supplement (Thermo-Fisher, 2%); and D-Glucose (Thermo-Fisher, 0.225 mg/mL). Neurons were grown in culture for 2 days, with a single media change at 1 day post-dissection. A subset of samples were treated with carbonyl cyanide m-chlorophenyl hydrazone (CCCP), a protonophore that induces apoptosis for 24 hours, on the second day post-dissection. After treatment, CCCP- treated cells and all other neurons on organic semiconductor films were fixed using 4% paraformaldehyde for 15 minutes and washed with phosphate-buffered saline (PBS). CCCP-treated cells were used as a control for TUNEL assay (data not shown).

Assessment of biocompatability

Neurons were labelled with Microtubule Associated Protein 2 (MAP2) and cell viability was determined using a TUNEL assay using a commercially obtained kit (Abcam). In the first instance, cultured neurons were incubated overnight with a chicken antibody to the neuronal protein MAP2 (1:500; ab5392, Abcam). After washing the primary antibody from the coverslips with PBS, the TUNEL assay was done. In brief, broken or fragmented DNA, indicative of cellular apoptosis, was labelled with 5-bromo-2′-deoxyuridine 5′-triphosphate (Br-dUTP) with a terminal deoxynucleotide transferase enzyme. At the same time as the TUNEL assay, neurons were incubated with secondary antibody against MAP2 (donkey anti-chicken FITC (490/525nm); 1:100, 703-095-155, Jackson Immuno Research) for one hour. Cells were washed with PBS and incubated with an anti-Br-dUTP antibody conjugated to a red fluorophore (excitation/emission: 488/576nm) for 30 mins. Cells were then washed and

counterstained with the blue nuclear label DAPI (358/461nm). Neurons were visualised with epifluorescenc microscopy (Nikon Eclipse 80*i*). Images of cultured cells were taken with a Nikon DS-Fi1 camera, and processed using Fiji software (National Institutes of Health). Supplementary Figure 1 shows good cell growth with minimal apoptosis compared to the control samples, confirming the biocompatibility of these semiconducting materials.

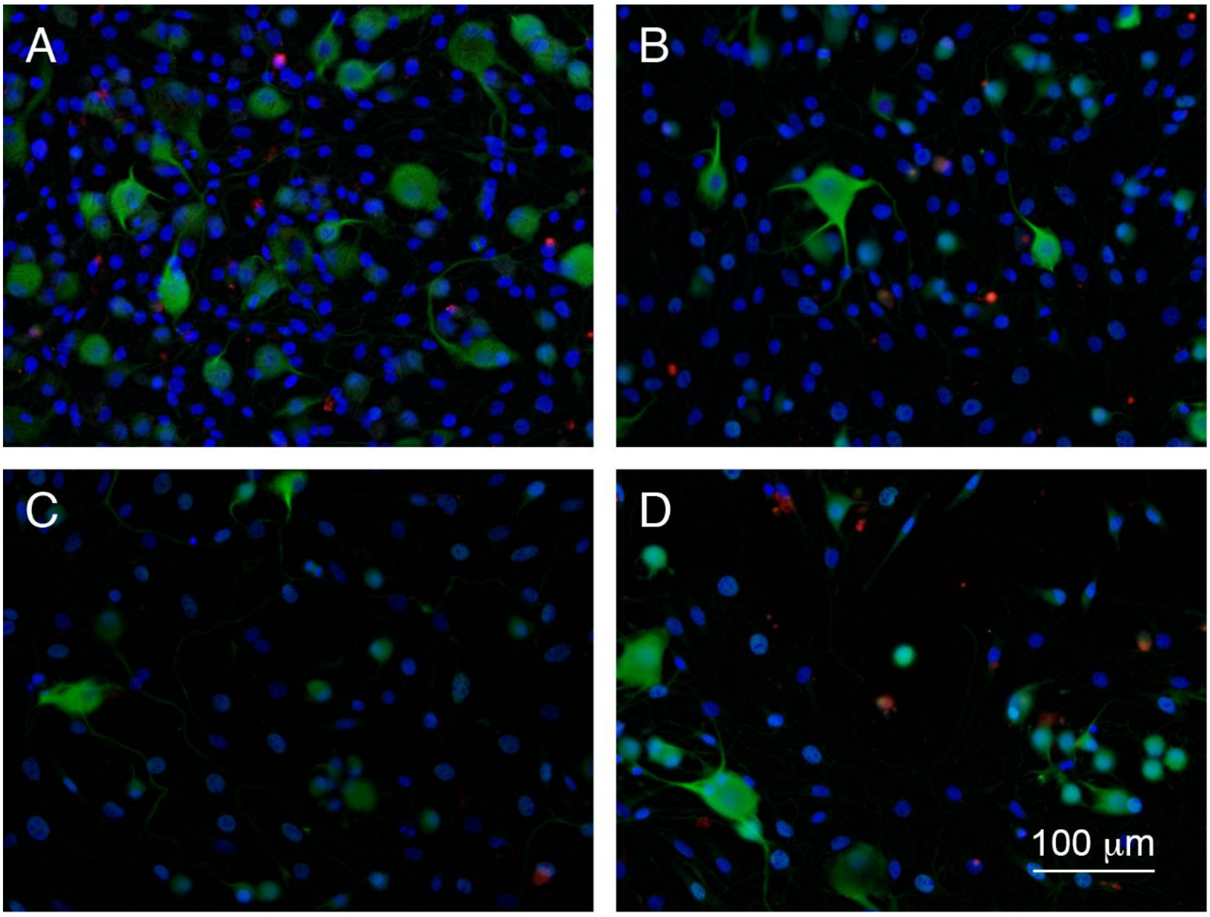

Supplementary Figure 1. Dorsal root ganglion neurons were cultured for 2 days on organic semiconductor films; (A) violanthrone 79, (B) TQ1, (C) C_{60} and (D) glass coverslips. Neurons are labelled with neuronal marker, microtubule associated protein 2 (MAP2, green). Broken or damaged DNA, indicative of cell death and apoptosis (red), was labelled with a TUNEL assay. Cell nuclei are labelled with 4′,6-diamidino-2-phenylindole (DAPI, blue). Scale bars represent 100µm.
